# A Case of Miller Fisher Syndrome and Literature Review

**DOI:** 10.7759/cureus.1048

**Published:** 2017-02-22

**Authors:** Sumera Bukhari, Javier Taboada

**Affiliations:** 1 Internal Medicine, Seton Hall University-St. Francis Medical Center, Trenton, NJ; 2 Neurology, Seton Hall University-St. Francis Medical Center, Trenton, NJ

**Keywords:** diplopia, ataxia, ophthalmoplegia, miller fisher syndrome, areflexia, double vision, muscular weakness, unsteady gait, guillain-barré syndrome

## Abstract

Miller Fisher syndrome (MFS)  was first recognized by James Collier in 1932 as a clinical triad of ataxia, areflexia, and ophthalmoplegia. Later, it was described in 1956 by Charles Miller Fisher as a possible variant of Guillain-Barré syndrome (GBS). Here, we write a case of a patient with atypical presentation of this clinical triad as the patient presented with double vision initially due to unilateral ocular involvement that progressed to bilateral ophthalmoplegia. He developed weakness of the lower extremities and areflexia subsequently. A diagnosis of MFS was made due to the clinical presentation and the presence of albuminocytologic dissociation in the cerebrospinal fluid (CSF) along with normal results of brain imaging and blood workup. The patient received intravenous immune globulin (IVIG), and his symptoms improved. The initial diagnosis of MFS is based on the clinical presentation and is confirmed by cerebral spinal fluid analysis and clinical neurophysiology studies. This case which emphasizes the knowledge of a rare syndrome can help narrow down the differentials to act promptly and appropriately manage such patients.

## Introduction

Miller Fisher syndrome (MFS), also called Fisher’s syndrome, was first recognized by James Collier in 1932 as a separate clinical triad of ophthalmoplegia, ataxia, and areflexia. Later, MFS was named after Charles Miller Fisher who reported it in 1956 as a limited variant of Guillain-Barré syndrome (GBS) [1]. MFS usually begins over days with the rapid development of three problems constituting the clinical triad: 1) ophthalmoplegia: weakness of the muscles within or surrounding the eye, 2) ataxia: the presence of abnormal, uncoordinated movements and poor balance with clumsy walking, and 3) areflexia: loss of deep tendon reflexes on physical examination. Patients typically get medical attention because of a rapid decrease in vision over days and difficulty walking. Here, we discuss the case of a patient with a clinical diagnosis of possible MFS and the approach to differential diagnosis concisely. Informed consent was obtained from the patient for this study.

## Case presentation

A 46-year-old male came to the emergency room (ER) complaining of double vision and difficulty walking. The double vision started five days prior, followed by weakness in both the legs leading to a wobbly gait. The patient was evaluated by the neurology team in the ER. The patient had a mild upper respiratory infection about ten days before the symptoms started. He denied any diarrhea or tick bite. He had no urinary/bowel incontinence. His past medical history was significant for asthma that was well controlled by albuterol inhaler as needed. He had no surgeries in the past. The family history was non-contributory. He denied smoking, alcohol use, or drug abuse. He took no medication except the albuterol inhaler as needed. He had no occupational exposure to neurotoxins.

On physical examination, his vital signs were within the normal range. He was fully alert and oriented to time, place, and person with an intact memory. There was paralysis of left eye abduction on cranial nerve examination. Both pupils were round, equal, and slowly reactive to light. No facial muscle asymmetry was noticed, with no evidence of seventh cranial nerve deficit. The muscle stretch reflexes were 1+ in all four extremities, and the plantar response was flexor. The Romberg test was negative. There was no focal sensory or motor deficit in the coordination of upper limbs. The heel-knee-shin test was mildly impaired with gait unsteadiness. The unsteady gait was demonstrated regardless of diplopia. An ophthalmology evaluation did not reveal the visual and retinal change. Next day, the patient complained of worsening of all symptoms and had total bilateral external ophthalmoplegia and sluggish pupillary light reflex. The muscle stretch reflexes became 0+ in the bilateral lower extremities and remained so until discharge.

The laboratory testing revealed normal complete blood count, comprehensive metabolic panel, thyroid stimulating hormone, and cardiac markers. The urine toxicology screens were negative for common substances of abuse. The serum alcohol level was normal. The electrocardiogram was normal. The erythrocyte sedimentation rate was 4 mm/hour. The serum anti-cholinesterase antibody test results were negative, which eventually refuted myasthenia gravis as a diagnosis. The chest radiograph showed no evidence of acute or chronic disease processes. The carotid Doppler did not show any sign of carotid stenosis. Computed tomography (CT) and magnetic resonance imaging (MRI) of the brain demonstrated no mass effect or evidence of an acute infarct. The serum glucose was 102 mg/dl, and the hemoglobin A1c was normal. The rapid plasma reagin for syphilis was negative. The serum antineutrophil cytoplasmic (ANCA) IgG titer was within normal reference range. The serum Lyme titer and Epstein-Barr virus (EBV) antibody titers were within the normal reference range. Aldolase was 8.2 units/liter (within normal range). A lumbar puncture was done, and the cerebrospinal fluid (CSF) analysis showed protein 43 mg/dl, glucose 66 mg/dl, white blood Cells 0/ul, red blood cells 0/ul, the presence of oligoclonal bands and negative gram stain and cultures. The electromyography (EMG) was normal. The test for anti-GQ1b antibodies was not done as the clinical presentation was classical for the diagnosis.

The patient was given steroids in the ER and subsequently treated with intravenous immune globulin (IVIG), and significant clinical improvement was noticed. Physical and speech therapy specialists worked with the patient on a daily basis. He was able to ambulate with some assistance. He was discharged on the 10th day of hospitalization to a subacute rehabilitation facility.

## Discussion

MFS accounts for one to five percent of all GBS cases in Western countries, but 19% and 25% in Taiwan and Japan, respectively [2]. MFS is twice as common in men than women [2]. It affects people of all ages, with the median age of onset being in the fifth decade [3]. MFS presents commonly with diplopia (78%), ataxia (48%), and both (34%). The less frequent symptoms consist of limb dysesthesia; blepharoptosis; face, bulbar, and pupillary palsies; mild (grade 4) motor weakness; and micturition disturbance [2]. These clinical signs are preceded by signs of upper respiratory tract infection in 56–76% of the patients. The most common pathogens are Campylobacter jejuni and Haemophilus influenzae. However, Mycoplasma pneumoniae and cytomegalovirus are also found to be associated [2, 4]. MFS onset is typically acute, beginning with neurologic symptoms approximately 8–10 days (range of 1–30), following the antecedent illness [2, 4-5]. The disease then progresses until a clinical nadir is reached approximately a week (range of 2–21) after the initial neurologic symptoms [2]. A diagnosis of MFS can be made with compatible clinical history taking, cardinal symptoms, normal findings on CT or MRI, and presence of albuminocytologic dissociation in the CSF of affected patients. Anti-GQ1b antibodies, which act against GQ1b (a ganglioside component of nerves), blocks acetylcholine release from the motor nerve terminals. It relates to the disease activity and can be used as a diagnostic marker in MFS [6]. It is not unique to MFS, but helps in serological confirmation to allow for more definite diagnostic certainty in the presence of confounding symptoms.

MFS is primarily a clinical diagnosis based on the key clinical presentation of ataxia, areflexia, and ophthalmoplegia. However, other neurological signs and symptoms may also be present leading to a more challenging diagnostic workup. Bilateral ophthalmoplegia is the first presenting feature of MFS. However in our case study, the patient noticed unilateral weakness which progressed to bilateral paralysis eventually. The rapid onset of eye symptoms in MFS can differentiate it from chronic diseases such as myasthenia gravis (MG), thyroid eye diseases (TEDs), and myotonic dystrophy (MD). In MG, ocular paralysis may switch from one eye to another and improve or worsen over the course of a day. MFS ophthalmoplegia progressively worsens until the nadir of symptoms has been reached and is complete before any recovery is noted [7]. TEDs may occur in patients already diagnosed with thyroid disease, or seldom it is the first presentation to get medical attention. TEDs include dry eyes, watery eyes, red eyes, bulging eyes, a "stare," double vision, difficulty closing the eyes, and problems with vision. However, our patient had no underlying thyroid disease or other clinical signs of TEDs. Ocular symptoms in MD are mainly characterized by variable ptosis, and in some cases, it progresses to complete paralysis. Other characteristic features of MD are weakness of the face, jaw, and neck as well as weakness of the extremities along with associated abnormalities of cardiac conduction defects, characteristic facies (long face with atrophy of temporalis and masseter), frontal balding, and variable intellectual impairment. The absence of most of these features makes the diagnosis of MD highly unlikely as in our case report.

It is clinically important to differentiate MFS from GBS as pure MFS is uncommon. Many MFS patients go on to develop the prominent, widespread weakness of GBS. It is noted that neurological deficit in MFS follows a descending pattern starting with external ophthalmalgia causing diplopia in the eyes. However, GBS characteristically presents with the ascending weakness or paralysis [2-3]. MFS may also be mistaken for an acute stroke. Cerebellar ischemia presents with an unsteady gait, dizziness, headache, eye movement dysfunction, as well as nausea and vomiting [8]. Though both MFS and ischemic event are acute, ataxic patients with cerebellar involvement show lateralization of ataxia while MSF patients typically lack lateralization of ataxia. It helps to differentiate MFS from most cerebellar lesions [8]. However, ataxia can also be seen in conditions involving spinocerebellar tracts, or the proprioception channels in peripheral nerves and dorsal columns. Toxins and medication like sodium channel modulators such as phenytoin and some chemotherapeutic agents such as fluorouracil can precipitate ataxic episodes. Ataxia secondary to alcoholic intoxication (drunken gait), mostly affects the lower extremities and is also associated with poor motor control of the hands, slurred speech, and impaired vision. It can be differentiated from ataxia in MSF patients as the progression of weakness follows a “top to down” fashion. In our case study, the brain imaging was negative for stroke, alcohol level was normal, and there was no exposure to such medication or toxins.

Areflexia can be present in certain neurological conditions damaging lower motor neurons in the spinal cord or peripheral nerves. Diabetes and vitamin B12 deficiencies can cause peripheral neuropathies and subsequently can present as areflexia. The anterior horn cell destruction, viewed in amyotrophic lateral sclerosis (ALS) and polio can present as areflexia. Paradoxically, areflexia can also occur in the subacute phase of spinal shock—seen in transection or compression of the spinal cord—which progresses to hyperreflexia as the pathology evolves [9]. Areflexia can also be seen in metabolic abnormalities like hypomagnesemia, alcoholism, and use of certain medication like antidepressants. In this case, the patient had a normal metabolic panel, no vitamin deficiencies or use of antidepressants, and no signs of spinal cord destruction.

MFS is a self-limiting condition and gradual improvement marks its recovery period and often the resolution of symptoms. Rarely, serious complications such as cardiac arrhythmia or respiratory failure have been reported [4]. Ataxia and ophthalmoplegia typically resolve within one to three months after onset, and near complete recovery is expected within six months [2]. Though areflexia may persist, it is not associated with functional disability. Although MFS follows a self-limiting course, immunomodulatory therapies including intravenous immune globulin and plasmapheresis have been used to hasten time to disease recovery and perhaps decreased a likelihood of progression to more severe conditions like GBS [10].

## Conclusions

Although MFS is a rare clinical entity to come across in the field of medicine, it holds a clinical significance due to the list of differential diagnosis. The presenting symptoms of ataxia and ophthalmoplegia may confuse the clinician and suggest an upper motor neuron sign or central cause. The presence of additional neurological symptoms may confound the clinical diagnosis. However, thorough neurological evaluation and the knowledge of such a rare syndrome can help to narrow down the differentials. This case emphasizes the atypical presentation of MFS as unilateral ocular paralysis progressing to bilateral disease, which can mislead the actual diagnosis. It also signifies the importance of history taking and clinical examination as the diagnosis of MFS is mostly a clinical diagnosis of exclusion of other possible differentials.
